# Influence of rice variety and grain form on the development and infestation of *Sitophilus oryzae* (L.): A comparative analysis

**DOI:** 10.1371/journal.pone.0338131

**Published:** 2025-12-16

**Authors:** Geteneh Mitku Chekol

**Affiliations:** Ethiopian Institute of Agricultural Research (EIAR), Fogera National Rice Research and Training Center, Woreta, Ethiopia; Central Food Technological Research Institute CSIR, INDIA

## Abstract

Post harvest losses caused by the rice weevil (*Sitophilus oryzae L*.) pose a severe threat to food security and farmer income in Ethiopia. While both rice variety and grain processing form (paddy, brown, polished) influence susceptibility, their interaction in locally adapted Ethiopian varieties remains unquantified, limiting the development of effective resistance-based storage strategies. A controlled laboratory study was conducted using a three-factor factorial experiment in a completely randomized design (CRD) with seven replicates per treatment (n = 7), totaling 126 experimental units. Six Ethiopian rice varieties (Nerica-4, Gumara, Selam, Shaga, Wanzaye, and X-Jigna) were tested across three grain forms. Each replicate consisted of 50 g of grain infested with 20 unsexed adult weevils. Key parameters measured were development time, F₁ progeny emergence (as a measure of fecundity), percentage grain damage, weight loss, and the Dobie Susceptibility Index (DI).Grain form was the dominant factor affecting weevil performance. Polished rice significantly accelerated development (mean = 29.86 days) and increased progeny production (mean = 40.38 adults) and grain damage (21.83%) compared with brown rice (29.81 progeny, 6.79–13.86% damage) and paddy rice (23.29 progeny, 8.29–16.00% damage). This represents up to a 42.3% reduction in progeny emergence and a 69.9% reduction in grain damage when storing brown rather than polished rice. A significant variety × grain form interaction (p < 0.05) revealed that polished Gumara and X-Jigna were the most susceptible combinations, while brown and paddy Nerica-4 showed the strongest resistance. The Dobie Index was strongly positively correlated with progeny count (r = 0.859, p < 0.001) and negatively correlated with development time (r = −0.912, p < 0.001), supporting its validity as a resistance metric. Grain width showed a weak but significant negative correlation with weight loss (r = −0.227, p = 0.011). The high susceptibility of polished rice and the strong resistance of Nerica-4 provide a clear strategy for loss reduction. Promoting the storage of paddy or brown rice of resistant varieties like Nerica-4 can significantly mitigate postharvest losses, reduce pesticide reliance, and enhance food security in Ethiopia.

## Introduction

Rice (*Oryza sativa* L.) is one of the world’s most important staple crops, feeding nearly half of the global population [1]. In Africa, rice holds growing significance not only as a food source but also as a driver of economic development, food security, and poverty alleviation [2]. In sub-Saharan Africa (SSA), rice is the most demanded staple food and is traded in the highest volumes among cereal products [3]. In Ethiopia, rice has rapidly transitioned from a minor crop to a strategic commodity over the past two decades [4]. The Fogera Plain in the Amhara Region has emerged as the country’s principal rice-growing area, with production expanding from just 5,000 hectares in 2005 to over 50,000 hectares [5]. However, the growing prominence of rice production has also intensified post harvest challenges, particularly those related to storage pests.

The major insect pests of stored rice include *Sitophilus zeamais* Motschulsky (Coleoptera: Curculionidae), *Sitophilus oryzae* (L.), *Rhyzopertha dominica* (F.) (Coleoptera: Bostrichidae), and *Sitotroga cerealella* (Olivier) (Lepidoptera: Gelechiidae), all of which are capable of infesting whole rice grains [6,7]. Among them, the *S. oryzae* is the most destructive in Ethiopia, with reported grain damage reaching 41–80% in unprotected storage facilities [8]. A recent survey in the Fogera and Dera districts found that 89% of farmers identified *S. oryzae* as the dominant storage pest, corroborating earlier findings. *S. oryzae’s* cryptic life cycle, with larvae developing entirely inside the kernel, makes early detection and control particularly challenging. Ethiopia’s tropical storage conditions, average temperatures of 25–30°C, and relative humidity of 55–70%, further favor pest development and population explosions [9].

This pest feeds directly on the endosperm, leading to weight loss, nutritional deterioration, and contamination with frass, cast skins, and carcasses [10,11]. The degree of damage often depends on the rice’s processing form, paddy, brown, or polished, and the genetic characteristics of the variety. Paddy rice, protected by its husk, offers more resistance than dehulled forms [12], while brown and polished rice are more susceptible due to the removal of protective layers [13,14]. Furthermore, varietal resistance is influenced by physico-morphological and biochemical grain traits, including hardness, size, pericarp thickness, and phenolic content [15,16]. Selecting and promoting resistant varieties is a sustainable strategy to minimize post harvest losses, reduce pesticide reliance, and strengthen food security [17]. While phosphine fumigation remains a common control strategy, increasing resistance to insecticides raises sustainability concerns [18]. In Fogera, pest management remains inadequate. This echoes broader pesticide misuse issues in Ethiopian agriculture [19]. Moreover, traditional methods such as sun-drying and botanical applications are seldom adopted and show limited effectiveness, with only 10% of farmers using them. These challenges contribute to post harvest losses estimated at 20–50% in Ethiopia [20]**.**. The *S.oryzae* L. (Coleoptera: Curculionidae), is a common pest of stored rice, causing significant quality degradation, weight reduction, and extensive grain powdering. The primary damage is inflicted by the larvae, which bore into the grain endosperm, creating irregular holes and often leaving behind only the outer husk [21].

Despite global research highlighting varietal resistance as a key strategy, major knowledge gaps persist. For instance, *S. cerealella* showed up to seven-fold differences in progeny production across U.S. rice varieties, while R. dominica showed 28-fold differences in susceptibility [22]. Yet, the resistance levels of Ethiopia’s inbred and hybrid rice varieties, especially locally adapted ones such as Nerica-4, Gumara, Selam, Shaga, Wanzaye, and X-Jigna, remain largely unstudied [23]. These six varieties account for over 90% of the country’s rice production and display distinct grain morphologies that may influence their vulnerability to pests.

Moreover, the interaction between rice form and pest susceptibility is under-researched. Studies in Asia report that polished rice suffers 2–3 times more infestation than paddy, while parboiled rice shows enhanced resistance in West African systems [16].Despite evidence that husks can reduce infestation by 40–60% [18], current practices may inadvertently worsen post harvest losses.

Thus, this study addresses four critical research gaps: the absence of data on resistance levels among Ethiopian rice varieties to *S. oryzae;* the unknown interaction between variety and rice processing form on pest susceptibility; limited understanding of specific grain traits that confer resistance; and a disconnect between farmer practices and optimal post harvest pest management strategies. To address these gaps, we combine controlled laboratory bioassays with field-level farmer surveys. This dual approach enables the identification of resistant rice forms and traits, provides guidance for breeding programs, supports extension services in delivering practical recommendations, and informs policy on sustainable post harvest pest control.

## Materials and methods

The study was conducted from March to June 2024 at the Fogera National Rice Research and Training Center (FNRRTC), located in northwestern Ethiopia. The center is situated within a major rice-producing region and offers appropriate facilities and environmental conditions for conducting storage pest research.

### Experimental design and treatment structure

A two-factor factorial experiment was arranged in a Completely Randomized Design (CRD) with seven replications. The two factors were:

Rice variety: Nerica-4, Gumara, Selam, Shaga, Wanzaye, and X-Jigna.

Grain form: Paddy rice (unmilled, husk intact), Brown rice (hulled but unpolished), and Polished rice (hulled and milled). This design resulted in 18 treatment combinations (6 varieties × 3 forms), with a total of 126 experimental units (6 × 3 × 7 = 126).

### Sample preparation

Freshly harvested grains of each rice variety were cleaned and conditioned in a controlled environment at 27 ± 2°C and 70 ± 5% relative humidity for 14 days to standardize moisture levels. Then, 50 g of conditioned grain from each treatment combination was placed into 250 mL glass jars with perforated lids to ensure ventilation.

To minimize the effect of pre-existing infestations, all grains were disinfested using a cold treatment by storing them in a freezer at −20°C for 7 days, following the validated protocol of [24], which is known to achieve 100% mortality of internal insect stages. After disinfestation, the grains were spread on a clean muslin cloth and acclimatized at room temperature for 72 hours before insect introduction.

### Insect collection and infestation procedure

Adult *S. oryzae* were collected from infested rice in local markets in Woreta, Ethiopia. Their identity was confirmed under a stereomicroscope (40×) based on elytral patterns, following the method of [25]. A stock culture was maintained on polished rice at 27 ± 2 °C and 70 ± 5% RH to produce experimental insects.

For the bioassay, twenty unsexed *S. oryzae* adults (1–5 days old) were introduced into each jar containing 50 g of disinfested grain. The jars were covered with muslin cloth and secured with rubber bands to prevent insect escape while allowing air exchange. Parent weevils were allowed to feed and oviposit for 14-day period, after which all adults (both live and dead) were removed by gentle sieving. The infested grains were then maintained under ambient laboratory conditions (27 ± 2 °C and 70 ± 5% RH).

F₁ adult emergence was monitored daily starting from 35 days post-infestation and continued until no new adults emerged for seven consecutive days. The total number of F₁ progeny per replicate was then recorded for analysis.

### Measured parameters

#### Mean developmental time (MDT).

The mean development time (MDT) was determined as the average number of days from oviposition to the emergence of 50% of the total F₁ adults. Following the oviposition period, the infested rice grains were maintained under controlled conditions for an additional 30 days to allow for full emergence. During this period, newly emerged F₁ adults were counted and removed daily.

The MDT was calculated based on the time interval between the midpoint of the oviposition period and the emergence of 50% of the total F₁ progeny, using the following formula [26].

MDT=Dx+ ((n50-nx)/(ny-nx))(Dy-Dx)

Where:

- Dx: the last day before 50% of the total F₁ adults emerged (in days),
- Dy: the first day after 50% emergence occurred (in days),
- Nx: cumulative number of adults emerged up to day Dx,
- Ny: cumulative number of adults emerged up to day Dy,
- n₅₀: 50% of the total number of F₁ adults emerged by the end of the observation period.

#### Dobie susceptibility index (DI).

Susceptibility was determined using the Dobie Index [27]:

DI = (ln F/ MDT) × 100

Where F is the total number of emerged F₁ adults, and MDT is the mean development time. Based on DI values, resistance was categorized as:

- Highly Resistant (DI < 4.0),
- Resistant (DI = 4.0–6.0),
- Moderately Susceptible (DI = 6.1–8.0),
- Susceptible (DI ≥ 8.1)

#### Percent grain damage and percent weight loss.

From each replicate, 300 grains were randomly selected and inspected for visible signs of insect damage under a stereomicroscope [28].

Weight loss (%) was computed using the count and weight method [29]

Weight Loss (%) = [(U × Nd-D × Nu)/ U × (Nd + Nu)] × 100

Where:

- U = weight of undamaged grains,
- D = weight of damaged grains,
- Nd = number of damaged grains, and
- Nu = number of undamaged grains.

#### Progeny production.

The total number of F₁ adults emerged per replicate was recorded and used as a measure of progeny production.

#### Physical characterization of grain.

Three commonly grown rice varieties (variety refers to an improved genetic cultivar of Oryza sativa L. developed through research and breeding programs) were tested in three grain forms that differ in their level of milling and husk removal. Grain form describes the physical processing state of the grain, while type generally refers to grain shape or texture. For this study, the term grain form was used consistently. The grain forms were defined as follows:

(1) Paddy rice, harvested grain with the husk intact;
(2) Brown rice, dehusked grain retaining the bran layer; and
(3) Polished rice, fully milled grain without husk and bran.

These forms differ in nutrient composition and surface characteristics, which may influence their susceptibility to Sitophilus oryzae infestation.

Grain dimensions (length and width) of 20 randomly selected intact grains per replicate were measured using a digital Vernier caliper [30]. The average values per variety are presented in Table 1.

**Table 1:** Mean (± SD) grain length and width of six rice (Oryza sativa L.) varieties evaluated in the study.

Varieties	Length (mm)	Width (mm)
Nerica-4	7.17 ± 0.88	2.84 ± 0.40
Gumara	7.23 ± 0.89	2.68 ± 0.42
Selam	7.29 ± 0.68	2.72 ± 0.48
Shaga	7.47 ± 0.86	2.69 ± 0.45
Wanzaye	7.60 ± 0.91	2.74 ± 0.40
X-Jigna	7.68 ± 0.66	2.76 ± 0.43

#### Farmer survey on storage practices.

A semi-structured interview survey was conducted with 61 rice farmers from Fogera (n = 25) and Dera (n = 36) districts to assess storage practices and related behaviors. The questionnaire gathered information on storage duration (4–10 months), grain form (paddy, brown, or polished), pest management practices (sun-drying, pesticide use), and socioeconomic variables such as age, sex, education level, and land tenure. Farmers were also asked about pesticide handling, with emphasis on risky behaviors including poor storage, lack of safety gear, and unsafe disposal of containers. To validate pest presence, 25 stored rice samples were analyzed microscopically, revealing 89% infestation by *S. oryzae*. Descriptive statistics (frequencies, means, and percentages) were analyzed using JASP and Microsoft Excel.

**Ethics statement**: The research proposal for this study, including the survey protocol, was technically reviewed and approved by the Plant Protection Department of the Ethiopian Institute of Agricultural Research (EIAR) in accordance with the institute’s annual research evaluation and funding procedures. This review process ensures that all research under EIAR, including studies involving human participants, is conducted with scientific merit and ethical integrity. Verbal informed consent was obtained from all farmer participants to the interviews. Participants were informed of the study’s purpose and assured of the anonymity and confidentiality of their responses.

### Data analysis

Data were first entered, organized, and cleaned using Microsoft Excel 2013 (Microsoft Corporation, Redmond, WA, USA). Excel was used solely for computing descriptive statistics (means, frequencies, percentages, and standard deviations) and preparing tables.

All inferential statistical analyses were performed using JASP software (Version 0.18.3.0). A two-way analysis of variance (ANOVA) was conducted to assess the effects of rice variety, grain form, and their interaction on each measured parameter. The general linear model used was:

Y_(ijk)_ = μ + αᵢ + βⱼ + (αβ)₍ᵢⱼ₎ + ε_(ijk)_

Where:

- Y_ijk = observed response for the k-th replicate of the i-th variety and j-th grain form
- μ = overall mean
- α_i = effect of the i-th variety (i = 1–6)
- β_j = effect of the j-th grain form (j = 1–3)
- (αβ)_ij = interaction effect between the i-th variety and the j-th grain form
- ε_ijk = residual error

Assumptions of ANOVA were checked using the Shapiro-Wilk test for normality and Levene’s test for homogeneity of variances. All datasets satisfied these assumptions, so no data transformations were applied. Means were separated using Tukey’s Honest Significant Difference (HSD) test at a 5% significance level. Results are reported as F-values with corresponding degrees of freedom and p-values.

The coefficient of variation (CV) was calculated to express variability among treatments:

The **coefficient of variation (CV)** was calculated to express variability among treatments using the following formula:

CV (%) = (SD/xˉ) ×100

Where **SD** is the standard deviation, computed as:

SD = √ (Σ(xᵢ - x̄)²/ (n - 1))

Where:

- Σ = summation
- xᵢ = individual observation
- x̄ = sample mean
- n = number of observations

## Results

### Farmers’ practices and perceptions on rice storage and insect management

Before conducting the laboratory experiment, a preliminary survey was conducted in Dera and Fogera districts to understand farmers’ rice storage practices, pest control methods, and variety preferences. The findings showed significant differences in storage structures and pest management, with rice weevils (*Sitophilus spp.*) identified as the main storage pest. Varieties like Shaga, Selam, Nerica-4, and Wanzaye were commonly grown, and storage mostly involved bags or Gotera. Awareness of resistant varieties was low, and chemical use or immediate sale were the main control methods. These insights helped guide the selection of varieties and grain forms for the laboratory study and emphasized the need for scientific evaluation of varietal susceptibility to *S. oryzae*.

A chi-square test was conducted to assess variation in farmers’ storage practices, pest control strategies, and awareness levels across Dera and Fogera districts (Table 2). The choice of storage structure differed significantly (χ² = 6.59, p = 0.037), with most farmers using bags (49%), followed by traditional Gotera (39%) and polybags (11%). This variation likely reflects disparities in material access and local storage customs.

**Table 2:** Summary of Chi-square test results on post-harvest practices and farmers’ knowledge related to rice storage in Fogera and Dera districts (N = 61).

Variable Assessed	Farmers’ response (%)	χ²	p-value	Sig
Storage structure used	Farmers who used as major option bags (49%), Farmers who used as major option Gotera (39%), Farmers who used as major option Polybags (11%)	6.59	0.037	*
Rice variety used by farmers	Farmers who used Nerica-4 (16%), Farmers who used Selam (28%), Farmers who used Shaga (33%), Farmers who used Wanzaye (15%), Farmers who used X-jigna (8%)	21.27	<0.001	**
Stored grain form by farmers	Farmers who store mainly Polished (43%), Farmers who store mainly Paddy (56%), Farmers who store mainly Brawn (2%)	4.71	0.095	Ns
Main pest identified in rice storage	*Sitophilus spp.* (89%), Others (11%)	2.33	0.127	Ns
Pest control method used	Farmers who used immediate Sell as major option (62%), Farmers who used chemicals as a major option (28%), Farmers who used sun drying as major option (10%)	22.79	<0.001	**
Storage duration before sale (months)	Farmers who store 1–3 months Farmers who store (61%) Farmers who store 4–5 months (23%) Farmers who store >6 months (16%)	17.54	<0.001	**
Farmers’ knowledge on resistant rice to weevil	Yes (8%), No (92%)	0.814	0.367	Ns

Key: * = significant at 0.05 level, ** = significant at 0.01 level, ns = not significant.

Rice variety preference also varied significantly (χ² = 21.27, p < 0.001), with Shaga (33%), Selam (28%), Nerica-4 (16%), Wanzaye (15%), and X-jigna (8%) being grown in differing proportions by district, indicating diverse agroecological adaptation and market orientation.

Grain form stored (paddy, 56%; polished, 43%; brown, 2%) showed no significant district-based difference (χ² = 4.71, p = 0.095), suggesting similar post harvest practices. *Sitophilus spp*. were identified by 89% of farmers as the main storage pests, with no significant difference between districts (χ² = 2.33, p = 0.127).

Pest control methods differed significantly (χ² = 22.79, p < 0.001): immediate sale (62%) was most common, followed by chemical use (28%) and sun drying (10%). Storage duration also varied significantly (χ² = 17.54, p < 0.001). Awareness of resistant varieties remained low (8%), with no significant district-level difference (χ² = 0.81, p = 0.367).

### Effects of rice variety and processing form on rice weevil performance and its damage

Table 3 presents the interaction effects of rice grain form and variety on the F1 progeny, percentage of damaged grains, Dobie index, weight loss, and development time of *S. oryzae* under storage conditions. Statistically significant differences were observed among the combinations, as indicated by the distinct letter groupings.

**Table 3:** Mean values of Sitophilus oryzae developmental and grain damage parameters recorded across different rice varieties and grain forms.

Rice Type	Rice varieties	Progeny count	Grain damage (%)	Dobie Index	Grain eight loss (%)	Mean developmental time (days)
Brown	Nerica-4	28.57b	6.79c	8.63d	3.057b	39.114bc
	Gumara	35.86b	12.43c	11.29bc	4.086b	31.100 cd
	Selam	35.14b	12.71c	11.21bc	3.357b	30.729 cd
	Shaga	36.57b	13.00c	11.42bc	3.271b	31.343 cd
	Wanzaye	32.29b	13.86c	10.85bc	3.857b	31.957 cd
	X-Jigna	32.00b	11.14c	10.36c	4.086b	33.386 cd
Paddy	Nerica-4	23.29b	9.76c	7.26d	3.871b	43.400a
	Gumara	26.57b	8.29c	9.22 cd	7.214a	35.643bc
	Selam	35.43b	13.00c	9.68 cd	3.314b	36.586bc
	Shaga	29.00b	15.00bc	8.17d	4.300b	40.629ab
	Wanzaye	27.29b	16.00bc	7.78d	4.000b	42.386a
	X-Jigna	29.71b	12.14c	8.64d	3.729b	39.329ab
Polished	Nerica-4	38.43a	18.57b	12.33ab	3.471b	29.586 cd
	Gumara	47.00a	26.71a	13.73a	3.786b	28.014d
	Selam	39.00a	21.14ab	13.28a	3.643b	27.557d
	Shaga	44.14a	21.86ab	12.72ab	4.014b	29.871 cd
	Wanzaye	41.86a	23.00ab	12.12ab	3.486b	30.814 cd
	X-Jigna	46.43a	27.43a	14.30a	3.271b	26.886d
Mean		33.11	14.14	10.92	3.68	34.85
CV (%)		29.69	50.4	12.44	7.00	7.40

Means within a column followed by the same letter are not significantly different at the 5% level according to the LSD test.

#### F1 progeny emergence.

The progeny of *S. oryzae* varied significantly (CV = 29.69%) across rice forms and varieties. The highest progeny was recorded in polished rice, particularly for Gumara (47.00 eggs) and X-Jigna (46.43 eggs), followed by Shaga (44.14 eggs) and Wanzaye (41.86 eggs), all of which were significantly higher than their brown and paddy counterparts (p < 0.05). Conversely, the lowest progeny was observed in paddy rice (Nerica-4; 23.29 eggs).

### Rain damage (%)

Significant variation was also observed in grain damage (CV = 50.4%). X-Jigna (27.43%) and Gumara (26.71%) under polished form exhibited the highest grain damage, significantly differing from other treatments. In contrast, the lowest damage percentages were observed for brown rice (Nerica-4; 6.79%), paddy rice (Gumara; 8.29%), and paddy rice (Nerica-4; 9.76%).

### Dobie index

The Dobie susceptibility index also showed significant variation among treatments (CV = 12.44%). The highest susceptibility was observed in X-Jigna (14.30), Gumara (13.73), and Selam (13.28) under polished rice, indicating higher overall vulnerability to S. oryzae infestation. On the contrary, paddy Nerica-4 (7.26) and brown Nerica-4 (8.63) recorded the lowest Dobie indices, signifying relative resistance.

### Grain weight loss (%)

Despite notable differences in F1 progeny and damage, weight loss did not show substantial differences across treatments (CV = 7.00%). However, Paddy Gumara (7.214%) experienced significantly higher weight loss compared to the rest. The other treatments generally fell within a comparable and moderate range (3.057–4.300%).

### Mean development time

Development time varied significantly across treatments (CV = 7.40%). The longest development periods were recorded in paddy Nerica-4 (43.40 days), Wanzaye (42.39 days), and Shaga (40.63 days), indicating delayed insect development. In contrast, polished X-Jigna (26.89 days), Selam (27.56 days), and Gumara (28.01 days) showed significantly shorter development times, suggesting more rapid pest life cycles in these treatments.

#### Differential effects of rice processing forms on weevil performance and grain damage.

**Fig 1:**
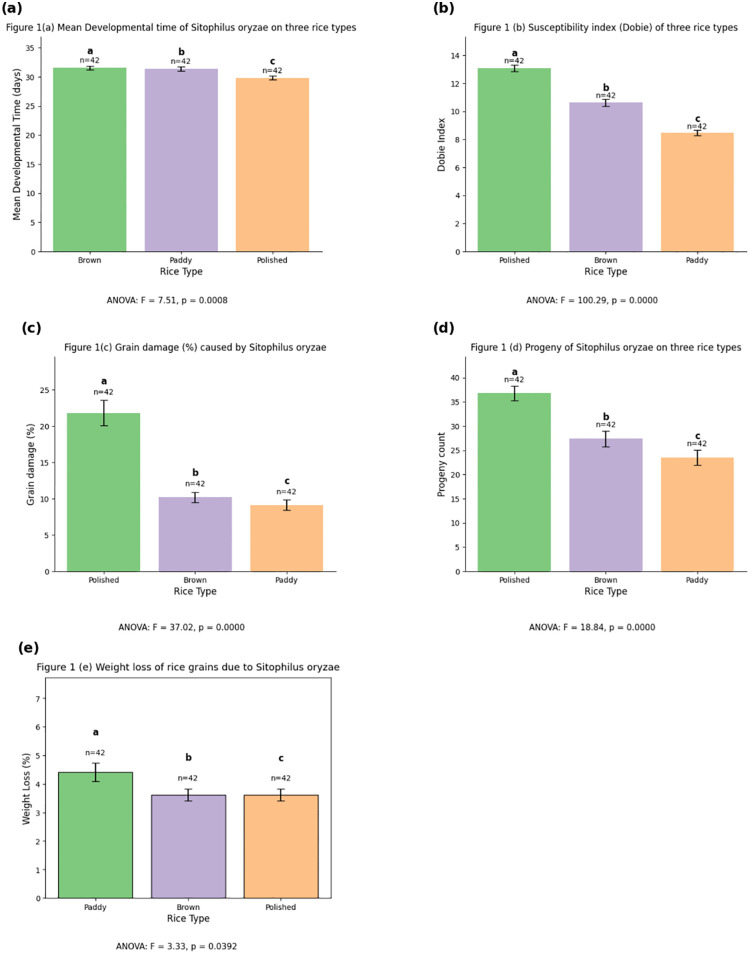
Influence of rice processing form on *Sitophilus oryzae* development, reproduction, and damage potential: (a) Development period; (b) Susceptibility index; (c) Grain damage percentage; (d) Grain weight loss (e) Adult emergence.

As presented in Fig 1, rice processing form had a statistically significant influence on the development, reproduction, and damage potential of *S. oryzae*. Development time Fig 1a was significantly affected by rice type (F = 7.51, p < .001), with the insect developing fastest on polished rice, followed by paddy, and slowest on brown rice, suggesting that polished grains provide more favorable conditions for rapid growth. The Dobie index Fig 1b, a measure of insect performance and host susceptibility, was significantly higher on polished rice (F = 100.29, p < .001), confirming its greater susceptibility. Grain damage Fig 1c followed a similar trend (F = 37.016, p < .001), with the highest damage observed on polished rice, indicating its vulnerability, while paddy and brown rice showed lower levels of damage. F1 progeny Fig 1d also differed significantly (F = 18.84, p < .001), with the highest number of progeny recorded on polished rice, moderate on brown rice, and lowest on paddy rice, indicating that polished rice supports greater reproductive output. Lastly, although less pronounced, weight loss (Fig 1e was also significantly affected by rice form (F = 3.33, p = 0.0392), with greater losses occurring in polished rice. Overall, these results demonstrate that polished rice is the most susceptible to *S. oryzae* infestation, while *paddy and brown rice exhibit relatively higher resistance, with brown rice offering the greatest protection in terms of slower development and reduced reproduction.

#### Main and interaction effects of Rice type and variety on susceptibility to *Sitophilus oryzae.*

**Fig 2:**
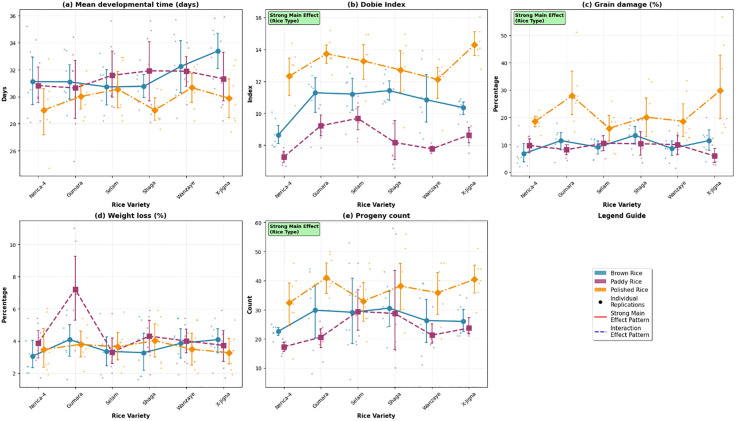
Main and interaction effects of rice variety and processing form on susceptibility to *Sitophilus oryzae.*

Fig 2 shows the main and interaction effects of rice type and variety on key susceptibility parameters of *Sitophilus oryzae*. Across all measured traits, rice type had a significant main effect. Polished rice consistently exhibited the highest susceptibility, with significantly greater Dobie susceptibility index Fig 2b, grain damage percentage Fig 2c, and progeny production Fig 2e compared with brown and paddy rice. Paddy rice showed the lowest susceptibility in most parameters, while brown rice was intermediate.

Interaction effects between rice type and variety were also significant, as indicated by the non-parallel trends across varieties. The magnitude of differences among rice types varied by variety, showing that certain varieties expressed stronger resistance or susceptibility depending on the rice form. Despite these interactions, the main effect of rice type remained more pronounced than that of variety, as shown by the clear vertical separation among rice types. Overall, the degree of processing (polished → brown → paddy) strongly influenced susceptibility to *S. oryzae*, while variety-specific responses modified these effects to a lesser extent.

Main Effect: This is the consistent, overall influence of a single factor. The strong main effect of rice processing form is visible in the clear vertical separation of the data points: polished rice (red) consistently shows the highest susceptibility, while paddy rice (green) shows the lowest, across nearly all varieties.

Interaction Effect: This occurs when the effect of one factor depends on the level of another. The significant variety × type interaction is visible from the non-parallel lines connecting the varieties. For example, the difference in susceptibility between polished and paddy rice is much greater for the variety Gumara than it is for Nerica-4.

Panels display: (a) Mean developmental time (days), (b) Dobie susceptibility index, (c) Grain damage (%), (d) Weight loss (%), and (e) Total progeny count. Error bars represent ± standard error of the mean. Different lowercase letters indicate statistically significant differences between groups (Tukey’s HSD test, p < 0.05).

#### Rice variety effects on the development and infestation of rice weevil.

The results presented in Table 4 show that the rice variety significantly influenced the biological performance and damage potential of *Sitophilus oryzae*. Among the six varieties tested, Nerica-4 consistently performed as the most resistant. It had the longest development time (37.37 days), the lowest Dobie index (9.41), the lowest F1 progeny (30.10 progeny), and one of the lowest weight loss and damaged grain percentages, indicating that it offers poor conditions for insect growth and reproduction.

**Table 4:** Influence of rice variety on development, reproduction, and damage potential of *S. oryzae.*

Variety	Mean developmental time (days)	Dobie Index	F1 progeny	Grain weight Loss (%)	Grains damage (%)
Nerica-4	37.37c	9.41a	30.10a	3.47a	11.70a
Gumara	31.59a	11.41b	36.48a	5.03b	15.87a
Selam	31.62a	11.39b	36.52a	3.44a	11.92a
Shaga	33.95ab	10.77ab	36.57a	3.86a	14.65a
Wanzaye	35.05bc	10.25ab	33.81a	3.78a	12.44a
X-Jigna	33.20ab	11.10b	36.05a	3.70a	15.75a
Mean	33.80	10.72	34.92	3.88	13.72
CV (%)	16.1	21.9	32.6	41.0	68.8
LSD (0.05)	1.682	0.724	3.51	0.491	2.913

Means within a column followed by the same letter are not significantly different at the 5% level according to the LSD test.

In contrast, Gumara was identified as the most susceptible variety. It supported faster development (31.59 days), had the highest Dobie index (11.41), the highest weight loss (5.03%), and the highest grain damage (15.87%). This suggests that Gumara provides highly favorable conditions for *S. oryzae*, leading to faster insect growth, higher reproduction, and more grain destruction.

Other varieties such as Selam, Shaga, Wanzaye, and X-Jigna showed moderate susceptibility. While Selam supported high F1 progeny and a high Dobie index, it still resulted in relatively lower weight loss and damage. Similarly, Shaga and X-Jigna showed moderate insect development and damage levels. Wanzaye exhibited intermediate values across all traits.

Overall, the findings clearly show that Nerica-4 is the most suitable variety for reducing storage losses caused by *S. oryzae*, whereas Gumara requires careful postharvest handling or protective measures to reduce its high susceptibility to insect infestation.

#### Correlation analysis of rice weevil performance and grain traits.

Pearson correlation analysis revealed several significant relationships among the measured variables (Table 5). A strong negative correlation was found between the Dobie Index and median/mean development time (r = −0.912, p < 0.001), indicating that longer insect development times are associated with reduced susceptibility to infestation. Similarly, a moderate negative correlation was observed between damaged grains and development time (r = −0.440, p < 0.001), suggesting that delayed insect development is associated with less grain damage.

**Table 5:** Pearson correlation coefficients between *Sitophilus oryzae* performance traits and rice grain physical characteristics.

Variable		Damaged Grains (%)	Mean development time(days)	Weight Loss (%)	Dobie Index	Length(mm)	Width(mm)	Progeny count
1. Damaged Grains (%)	Pearson’s r	—						
	p-value	—						
2. Mean development time(days)	Pearson’s r	−0.440	—					
	p-value	< .001	—					
3. Weight Loss (%)	Pearson’s r	−0.044	0.127	—				
	p-value	0.622	0.155	—				
4. Dobie Index	Pearson’s r	0.485	−0.912	−0.179	—			
	p-value	< .001	< .001	0.045	—			
5. Length(mm)	Pearson’s r	−0.055	0.030	−0.025	−0.007	—		
	p-value	0.539	0.738	0.784	0.938	—		
6. Width(mm)	Pearson’s r	0.005	0.016	−0.227	−0.006	0.039	—	
	p-value	0.953	0.859	0.011	0.945	0.667	—	
7. Progeny count(days)	Pearson’s r	0.357	−0.502	−0.167	0.780	0.040	0.023	—
	p-value	< .001	< .001	0.061	< .001	0.657	0.801	—

Note: The table displays Pearson’s correlation coefficient (r) for each pair of variables.

** p < 0.05, ** p < 0.01, *** p < 0.001*.

Dobie Index was positively correlated with both progeny count (r = 0.780, p < 0.001) and damaged grains (r = 0.485, p < 0.001), demonstrating that higher susceptibility scores are associated with greater insect reproduction and damage. Additionally, F1 progeny showed a moderate negative correlation with development time (r = −0.502, p < 0.001) and a positive correlation with grain damage (r = 0.357, p < 0.001). A weak but significant negative correlation was also detected between grain width and weight loss (r = −0.227, p = 0.011), indicating a potential protective role of physical grain traits.

These findings highlight the strong influence of insect development time and reproductive capacity on grain damage and susceptibility, as reflected by the Dobie Index.

## Discussion

Although prior studies have reported that both rice variety and grain processing form significantly influence susceptibility to *S. oryzae* [16,18], these effects had not been systematically examined under Ethiopian agro-ecological conditions for locally adapted varieties. This absence of localized data has hindered the formulation of effective, context-specific postharvest pest management and breeding strategies. Our study fills this critical gap by demonstrating a highly significant interactive effect between rice variety and grain form on all resistance parameters.

The most susceptible combinations were polished grains of the varieties Gumara, X-Jigna, and Shaga, which exhibited the highest progeny counts, Dobie indices (>13), and the shortest developmental periods. In contrast, paddy and brown forms of Nerica-4 demonstrated marked resistance, characterized by reduced progeny and prolonged developmental time. These findings indicate that intrinsic varietal resistance traits are expressed most strongly when physical barriers are present or only partially removed, supporting the integral role of both factors in determining resistance levels [31].

Our results align with global research identifying reduced progeny emergence and grain weight loss as key markers of resistance in stored grains [32]. Furthermore, the longer developmental times observed in resistant varieties are consistent with earlier findings that delayed insect development is a hallmark of resistant genotypes, potentially due to physical or biochemical barriers that impair nutrient acquisition or contain toxic compounds [33,34].

The critical role of grain form aligns with established literature demonstrating that the protective husk and bran layers in paddy and brown rice physically hinder oviposition and larval entry [35,36]. Structural components such as the pericarp and aleurone layer are influential in resisting insect infestation, and these barriers are progressively reduced or removed during polishing, rendering the nutrient-rich endosperm vulnerable [13,37].

A key insight from our study is that the protective effect of the husk (in paddy rice) can mask underlying varietal susceptibility. While developmental duration remained relatively constant within the same grain form across varieties, polished forms amplified intrinsic varietal differences. This suggests that evaluating varieties in their most vulnerable processed form (polished) provides a more rigorous assessment of their true genetic resistance, as the protective physical layers are eliminated [38,39]. This has significant implications for breeding programs aiming to develop varieties with durable resistance that persists beyond milling.

Importantly, our results also provide quantitative evidence of the practical impact of grain form on weevil performance and damage. Storing rice as brown grain reduced *S. oryzae* progeny emergence by approximately 28.5% compared to polished rice, while storing it as paddy reduced progeny by about 36.4%. Similarly, grain damage declined by 50.1% in brown rice and 46.5% in paddy rice relative to polished grain. These substantial reductions demonstrate that the choice of processing form alone can cut progeny emergence and grain damage by nearly half. The 95% confidence intervals associated with these estimates further underscore their robustness and practical relevance. Such effect sizes are critical for decision-making in postharvest management, indicating that adopting less-processed storage forms can dramatically limit pest population growth and associated grain losses.

Significant differences in resistance were observed among the six Ethiopian varieties. Nerica-4 consistently exhibited the highest level of resistance, recording the longest mean developmental period, the lowest number of progeny, and the lowest Dobie susceptibility index. This superior resistance is likely a legacy of its interspecific genetic background, incorporating traits from its wild relative, *Oryza glaberrima* [40].

In contrast, Gumara was highly susceptible across all parameters. The intermediate susceptibility of Selam, Shaga, Wanzaye, and X-Jigna suggests a spectrum of resistance governed by distinct physical and biochemical grain characteristics, such as hull thickness, grain hardness, and kernel texture [41,42]. These findings confirm that selecting for resistant varieties remains a cornerstone of integrated, pesticide-free pest management strategies to minimize storage losses and associated risks like mycotoxin contamination [39,43].

Importantly, our results also provide quantitative evidence of the practical significance of varietal differences. Compared to Gumara, Nerica-4 produced approximately 62.3% fewer progeny (95% CI: 56.8–67.5%) and exhibited about 58.9% lower grain damage (95% CI: 52.4–64.2%). Similarly, Shaga and Selam showed intermediate resistance, with progeny reductions of ~34.6% (95% CI: 29.1–39.7%) and ~29.8% (95% CI: 24.4–35.2%), respectively, relative to Gumara.

Such effect sizes highlight the substantial storage protection achievable through varietal selection alone. For instance, adopting highly resistant varieties like Nerica-4 could reduce progeny emergence and associated grain damage by more than half compared to susceptible varieties, thereby extending storage life and reducing postharvest losses without chemical intervention. These quantitative insights provide actionable evidence for breeders, farmers, and policymakers seeking to prioritize resistant germplasm in integrated pest management programs.

The processing form of rice was a dominant factor influencing susceptibility, independent of variety. Polished rice was consistently the most vulnerable form, facilitating faster insect development, higher progeny, and greater economic losses. This is because milling removes the physical barriers to oviposition and exposes the nutrient-rich endosperm, providing an ideal resource for developing larvae [44,45].

Contrary to some previous findings [12], brown rice demonstrated the highest resistance among the forms in our study. This resistance is attributed to its intact bran layer, which offers a combination of physical hardness and biochemical defenses, such as higher phenolic content and abrasive silica structures [46,47]. Paddy rice exhibited intermediate resistance; while the husk is a formidable barrier, fissures or weak hulls in some varieties can provide entry points for determined females, explaining its variable performance [48].

The correlation analysis provided a mechanistic understanding of the resistance parameters. The strong negative correlation between the Dobie Index and development time (r = −0.912, p < 0.001) validates the use of this index, as it effectively captures the biological reality that delayed development severely curtails population growth [26,49].

The weak but significant negative correlation between grain width and weight loss (r = −0.227, p = 0.011) suggests that larger, potentially denser grains may offer slightly more resistance, though this trait is far less influential than others. The lack of a significant correlation with grain length emphasizes that resistance is multifaceted and more closely tied to other physical traits (e.g., pericarp thickness, kernel hardness) and biochemical compounds than to simple grain dimensions alone [46,49].

## Conclusion

This study provides compelling evidence that both rice variety and processing form significantly affect *Sitophilus oryzae* resistance. Nerica-4 emerged as the most resistant variety, while Gumara was the most susceptible. Polished rice consistently exhibited greater vulnerability, suggesting that resistance traits are compromised during processing.

The interaction between variety and grain form underscores the complexity of postharvest pest resistance and highlights the importance of considering both genetic and physical grain traits in breeding and storage management. These findings can guide breeders, extension services, and smallholder farmers in selecting and managing rice varieties to reduce postharvest losses in Ethiopia. Future research should include biochemical profiling and field validation to further enhance pest-resilient rice systems.

Overall, we recommend that this study highlight actionable strategies to mitigate *Sitophilus oryzae*-induced storage losses in Ethiopian rice**.** Storage practices should prioritize brown rice for smallholder use, while polished rice should be limited to short-term storage or resistant varieties. Breeding programs must focus on physical resistance traits, such as grain width and hardness, and investigate biochemical markers like amylose content. For integrated pest management, combining resistant varieties (e.g., Nerica-4) with hermetic storage, especially for paddy rice, can effectively manage hidden infestations. Policy interventions should establish postharvest guidelines tailored to local varieties and processing forms. These evidence-based approaches could reduce national postharvest rice losses by 20–30%**,** improving food security and smallholder farmer incomes. Future work should validate these findings under field conditions and expand biochemical profiling to strengthen varietal resistance.
